# Socioenvironmental Factors Influencing Distribution and Intensity of Soil-Transmitted Helminthiasis in the Brazilian Amazon: Challenges for the 2030 Agenda

**DOI:** 10.1155/2021/6610181

**Published:** 2021-02-04

**Authors:** Deiviane Aparecida Calegar, Polyanna Araújo Alves Bacelar, Brenda Bulsara Costa Evangelista, Kerla Joeline Lima Monteiro, Jéssica Pereira dos Santos, Mayron Morais Almeida, Márcio Neves Bóia, Filipe Anibal Carvalho-Costa

**Affiliations:** ^1^Laboratório de Epidemiologia e Sistemática Molecular, Instituto Oswaldo Cruz, Fundação Oswaldo Cruz, Rio de Janeiro, Brazil; ^2^Escritório Técnico Regional Fiocruz Piauí, Teresina, Piauí, Brazil; ^3^Laboratório de Biologia e Parasitologia de Mamíferos Silvestres Reservatórios, Instituto Oswaldo Cruz, Fundação Oswaldo Cruz, Rio de Janeiro, Brazil

## Abstract

Soil-transmitted helminthiasis (STHs) are poverty-related diseases with high prevalence rates in developing countries. The present study aims to describe the epidemiological scenario of STHs in an urban population in the Brazilian Amazon. A cross-sectional survey (*n* = 349 children aged 1–15 years) was carried out to obtain faecal samples and sociodemographic and sanitation data. Among the children, 143 (41%) were positive for at least one STH. Prevalence rates of infections by *A. lumbricoides*, *T. trichiura*, and hookworms were 24.4%, 42.6%, and 9%, respectively. A logistic regression multivariate model showed that infection with *A. lumbricoides* is significantly more frequent in children aged 11–15 years (odds ratio [OR] = 2.38; 95% confidence interval [CI] = 1.15–4.94; *p*=0.018) and the presence of latrines inside houses is a protection factor against ascariasis (OR = 0.38; 95% CI = 0.17–0.85; *p*=0.019). Positivity for *T. trichiura* is higher in the 5–10 (OR = 3.31; 95% IC = 1.85–5.89; *p*=0.001) and 11–15 age groups (OR = 3.16; 95% IC = 1.66–6.00; *p*=0.001), in children living in poor families (OR = 1.78; 95% IC = 1.01–3.14; *p*=0.045) and practicing open evacuation (OR = 2.07; 95% IC = 1.07–3.99; *p*=0.029). Hookworm infection is more frequent in children aged 11–15 years (OR = 6.70; 95% IC = 1.91–23.43; *p*=0.002), males (OR = 6.35; 95% IC = 2.00–20.14; *p*=0.002), and those living in stilt houses (OR = 3.52; 95% IC = 1.22–10.12; *p*=0.019). The use of albendazole in the last six months was a protection factor against hookworm infection (OR = 0.31; 95% IC = 0.10–0.96; *p*=0.042). The proportion of mild, moderate, and severe infections was 55.2%, 37.8%, and 7%, respectively, for *A. lumbricoides*, 72.4%, 24.3%, and 3.3% for *T. trichiura*, and 93.8%, 3.1%, and 3.1% for hookworms. Significantly higher worm burdens in *T. trichiura* and hookworm infections were associated with practicing open defecation and living in stilt houses. The data points to the need to improve sanitation infrastructure in Amazonian cities with similar sociodemographic and environmental characteristics.

## 1. Introduction

Ending extreme poverty, controlling neglected tropical diseases, and ensuring access to clean water and sanitation are interlinked sustainable development goals defined by the United Nations in the 2030 Agenda. Soil-transmitted helminthiasis (STHs) are poverty-related parasitic diseases infections with high prevalence rates in developing countries in Africa, Asia, and Latin America [1, 2]. STHs are caused by a group of enteric parasites whose biological cycle involves the passage of a developmental stage through the soil. This group includes *Ascaris lumbricoides*, *Trichuris trichiura*, and hookworms (*Necator americanus* and *Ancylostoma duodenale*). Transmission is, therefore, dependent on environmental contamination with faecal matter in inadequate sanitation infrastructure backgrounds [3].

Although STHs can frequently evolve in a relative host–parasite balance, infections with a high parasitic burden can cause acute and life-threatening complications, including intestinal obstruction and perforation by *A. lumbricoides*, dysentery and rectal prolapse by *T. trichiura*, and severe anaemia caused by hookworms [4–6]. High worm burdens are associated with reinfections due to constant exposure to environments contaminated with eggs (*A. lumbricoides* and *T. trichiura*) or larvae (hookworms) [7].

The last nationally based prevalence survey in Brazil was conducted from 2010 to 2015 with school children aged 7 to 17 years. It showed a wide regional variation in prevalence rates, with a strong correlation with the socioeconomic and development indicators of the states [8]. STHs prevalence rates are substantially higher in the Amazon region where a large proportion of the population lives in poverty and the sanitation and human development indexes (HDI) are worse than the average Brazilian standard [8]. In the Amazon region, demographic growth out in recent decades has not been accompanied by the implementation of sanitation infrastructure, enabling the spread of parasitic diseases [9–11]. The Amazon is also the Brazilian region with the highest prevalence of malnutrition in children [12, 13] and the municipalities with the highest proportion of population with inadequate sanitation systems.

STHs control policies have been strongly based on collective chemoprophylaxis (mass drug administration, MDA) with single doses of albendazole (400 mg), as recommended by the World Health Organization [14, 15]. In Brazil, this strategy has been encouraged in the last decade, with the distribution of albendazole in schools and health unities [16]. Nonetheless, aspects such as the emergence of parasite resistance to benzimidazole derivatives, as well as the effectiveness of these actions, through the assessment of cure rates and faecal egg count reduction rates of distinct STH species, are rarely considered [17–19].

Some issues not addressed in the last prevalence surveys in Brazil include the variations in parasitic loads (since quantitative analyses have not been carried out) and groups most vulnerable to infections. The present study describes the epidemiological picture of STHs in a typical urban population in the Brazilian Amazon.

## 2. Materials and Methods

### 2.1. Description of the Studied Area

**Figure 1:**
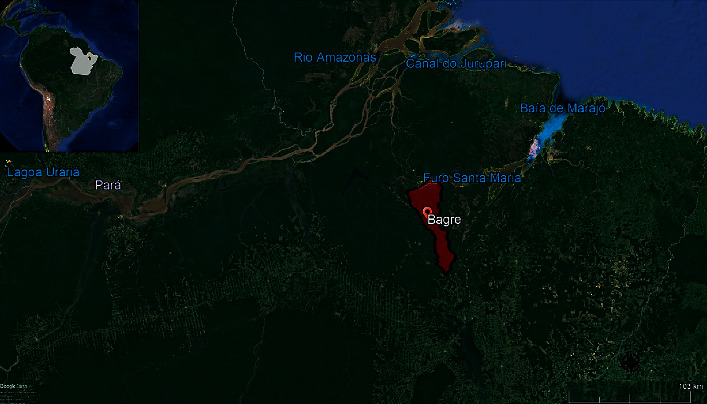
Map of the study area demonstrating the urban districts of Bagre and the Marajó Archipelago, Pará State, Brazilian Amazon.

The territory of the Marajo archipelago (Amazon River Delta, Para, Brazil), with 104,606.90 Km^2^ and 533,000 inhabitants, is divided into sixteen municipalities. The Portel microregion has four municipalities, including Bagre, where the study was conducted, which has an HDI of 0.471 (very low) and a population of 30,000 inhabitants. The municipality's urban area is located on one of the islands in the fluvial-maritime archipelago, with poor sanitary infrastructure and demographic concentration (Figure 1). There is no supply of drinking water and the population draws water from the river, adding, in the houses, sodium hypochlorite and aluminium sulphate to make it clear and suitable for consumption. Fishing and subsistence agriculture are the main economic activities. The municipality which has no sewage network and the waste is deposited in rudimentary pits or soil in the peridomestic environment. In the urban area of Bagre, a proportion of the population lives in a neighbourhood of stilt houses, in which domiciles have a closer contact with river waters.

### 2.2. Study Design, Sampling, and Recruitment

A cross-sectional survey was carried out in March 2020, to obtain faecal samples and sociodemographic and sanitation data. Faecal collectors were distributed by community health agents and 349 children were included. This sample size was obtained considering a population of 10,000 children [20], expected frequency of 35%, margin of error of 5%, and confidence level of 95%. When delivering faecal samples to the basic health units, those responsible for the children were face-to-face interviewed.

### 2.3. Parasitological Techniques

Faecal samples were collected in bottles without preservatives and sent within 24 hours to the laboratory set up at a local health unit. After preparing faecal suspensions, samples were examined by light microscopy using the Ritchie technique [21] and fluctuation in hypertonic glucose solution (Sheather technique) [22]. Positive samples were also analysed by the Kato-Katz [23] technique in order to determine the parasitic load, measured in eggs per gram of faeces (epg). The intensity of infections was determined as follows: (i) ascariasis, light in the range of 0–4,999 epg, moderate from 5,000 to 49,999 epg, and heavy with ≥50,000 epg, (ii) trichuriasis, light with a count ≤999 epg, moderate with a load between 1,000 and 9,999 epg, and severe with intensity ≥10,000 epg, and (iii) hookworm, mild with a parasitic load ≤1,999 epg, moderate between 2,000 and 3,999 epg, and heavy with ≥4,000 epg [24].

### 2.4. Assessment of Family Income, Demographic Characteristics, and Use of Albendazole in the Last Six Months

Those responsible for children were asked about all sources of family income; the values were added and divided by the number of family members to calculate the per capita monthly family income. Poverty was defined when this value was below R$ 132,00, which corresponds, roughly, to 26 USD (considering the exchange rate of 1 USD = R$ 5,21). Researchers also gathered information about the site of defecation, that is, if the family had a latrine inside the house and if members of the family practice open defecation in the peridomestic environment. The houses were also classified into stilt houses or ground houses. The utilization of antihelminthic drugs in the last six months was assessed, albendazole being the only drug cited by research subjects.

### 2.5. Statistical Analysis

Bi- and multivariate analyses (logistic regression) were performed, considering the positivity for the different species of STHs as dependent variables. The independent variables for the analyses were sex, age group, previous use of albendazole (in the last six months), living in poverty, living in stilt houses, practicing open defecation, having a latrine inside the house, and being infected with other STHs. Variables with associations generating *p* value ≤ −0.20 were selected to the multivariate model. The association between infections with distinct STHs was assessed in the bivariate analysis. The egg count medians and respective interquartile ranges were compared through a nonparametric test (Mann-Whitney). Statistical significance was used at *p* < 0.05 for bi- and multivariate analysis. Statistical analyses were performed with Epi Info 2000® (CDC, Atlanta, Georgia, USA).

### 2.6. Ethics

This study was approved by the Research Ethics Committee of Oswaldo Cruz Institute (IOC), license number 12125713.5.0000.5248. The consent form was completed by the guardians. Children and adolescents also provided an assent form.

## 3. Results

### 3.1. Prevalence, Distribution, and Factors Associated with Soil-Transmitted Helminthiasis

**Figure 2:**
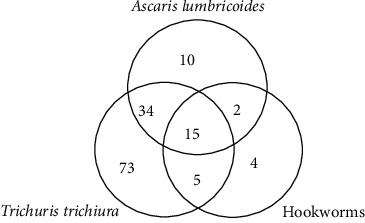
Diagram presenting the number of single and coinfections with distinct soil-transmitted helminths.

General prevalence rates of infections by *A. lumbricoides*, *T. trichiura*, and hookworms were 24.4%, 42.6%, and 9%, respectively. Among the faecal samples analysed, 143 (41%) were positive for at least one STH. The number of coinfections is depicted in Figure 2.

As shown in Tables 1–3, positivity for ascariasis, trichuriasis, and hookworm infection reached 31.4%, 53.9%, and 15.7%, respectively, in the 11–15-year-old age group, with positivity rates being significantly higher in this age group. The bivariate analyses demonstrated that open defecation and lack of a latrine inside the house were associated with ascariasis, trichuriasis, and hookworm infection. In addition, poverty was associated with trichuriasis and hookworm infection. Living in stilt houses was associated with hookworm infection. The logistic regression multivariate model showed that infection with *A. lumbricoides* is significantly more frequent in children aged 11–15 years and that the presence of latrines inside houses is a protection factor. Positivity for *T. trichiura* is higher in the age groups of 5–10 years and 11–15 years, in children living in poor families and practicing open evacuation. Hookworm infection is more frequent in children aged 11–15 years, males, and those living in stilt houses. The use of albendazole in the last six months was associated with a protection factor against hookworm infection.

### 3.2. Parasite Burden of Soil-Transmitted Helminths

**Figure 3:**
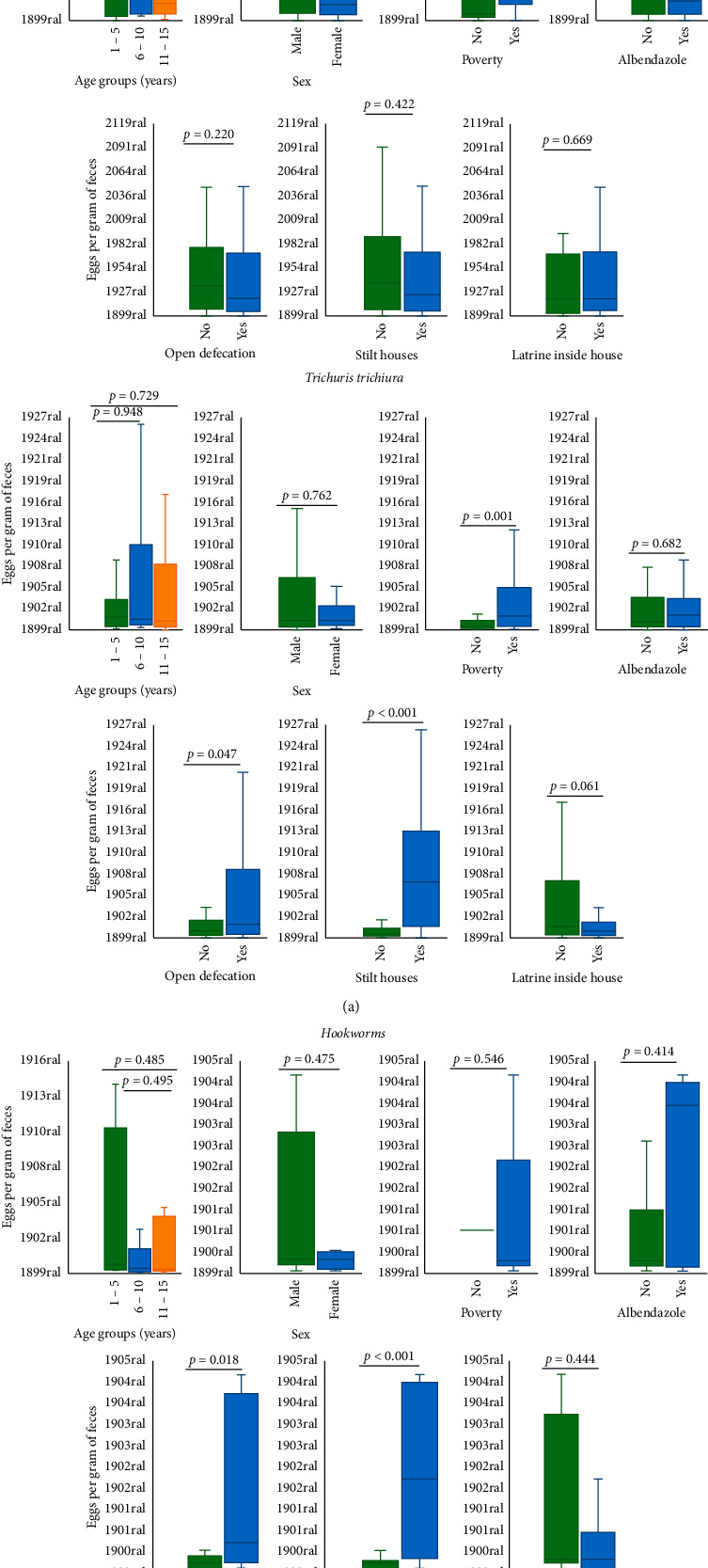
Boxplots depicting the comparisons of median and interquartile ranges of faecal soil-transmitted helminths egg counts in distinct groups defined by demographic and socioenvironmental characteristics. Comparisons were performed through the Mann–Whitney nonparametric test.

The frequency of mild, moderate, and severe infections was 55.2%, 37.8%, and 7%, respectively, for *A. lumbricoides*, 72.4%, 24.3%, and 3.3% for *T. trichiura*, and 93.8%, 3.1%, and 3.1% for hookworms. The graphs in Figure 3 show the comparison of medians and interquartile ranges of epg counts in distinct subgroups defined by independent variables. Among the positive children, significantly higher worm burdens in *T. trichiura* and hookworm infections were associated with practicing open defecation and living in stilt houses.

## 4. Discussion

The results demonstrate the endemicity of STHs in urban communities in Bagre, Brazilian Amazon. Data suggest that the current measures to control these infections in the region are inefficient. It can be inferred that the children in areas with the same socioenvironmental characteristics in this region are frequently affected by STHs.

The most prevalent STHs in the region are trichuriasis and ascariasis, emphasizing the first, which affects almost half of the children, with a much higher value than the countrywide prevalence rate, which is 5.4% [8]. Both infections are transmitted orally, and the results point to wide environmental contamination by faecal matter, in an unfavourable sanitation scenario.

Data from this study show that, even in an urban microregional scale, it is possible to identity a heterogeneous distribution of STHs. Therefore, it was demonstrated that socioenvironmental factors, including the infrastructure available to destination of faeces, which includes the availability of a latrine inside the house, and the necessity of practicing open defecation are determinants to disease production. In addition, among infected subjects, open defecation and living in stilt houses were associated with higher infection intensities for trichuriasis and hookworm infection.

The last national STHs survey carried out in Brazil showed that Amazonian states have prevalence rates above the national average [8]. Urbanization processes in the Amazon region often involve the demographic concentration of the population of Amerindian descent in a context of poverty and lack of health infrastructure [1, 25]. These demographic phenomena have created a favourable scenario for the spread of infectious diseases and the region where malaria is hyperendemic was also hit by a cholera epidemic in the 1990s [26]. The Marajó archipelago has cities with a very low HDI, and the region is marked by a high proportion of the population living in poverty. The region has a large water supply, but there is no infrastructure for treatment and the residents themselves process the water with hypochlorite and clarifiers/decanters such as aluminium sulphate. Storage is also done in precarious conditions, in improvised and reused containers, such as PET bottles.

MDA strategies seem to be ineffective in the region. It is likely that the administration of anthelmintics is performed with low frequency, there are constant reinfections, and the effectiveness of single dose of albendazole is low, which may point to parasite's emerging resistance to this drug. It was demonstrated that hookworm infections were significantly less frequent among children that received albendazole in the six months prior to the survey.

Coinfections were frequent among children who harbored STHs, with a high proportion of positives infected with more than one species. Therefore, an infection by one species was significantly associated with an infection with a second species, demonstrating the vulnerability of certain groups in the population studied.

Data from this study demonstrate that reducing the proportion of children living in poverty and increasing access to improved defecation sites can significantly reduce the prevalence and intensity of STHs, reducing the demand for collective deworming campaigns. Challenging scenarios are described so that the sustainable development goals can be locally achieved in the context of the United Nations 2030 Agenda. In conclusion, the study demonstrates the great vulnerability of Amazonian urban communities to faecal-borne diseases such as STHs. The data points to the need to optimize sanitation infrastructure in Amazonian cities with similar sociodemographic and environmental characteristics and high STH prevalence rates.

## 5. Conclusions

The study demonstrates the great vulnerability of Amazonian urban communities to faecal-borne diseases such as STHs. It is assumed that other cities with similar sociodemographic and environmental characteristics in the Marajó archipelago also have high STH prevalence rates. The data point to the need to implement control strategies, such as preventive chemoprophylaxis and improvement of health and sanitation infrastructure.

## Figures and Tables

**Table 1:** Factors associated with ascariasis in children living in the urban area of Bagre, Pará State, Brazilian Amazon.

	Positive for *A. lumbricoides*, *N* (%)	Crude OR (95% CI)	*p*-value	Adjusted OR (95% CI)	*p* value
*Age group (years)*					
0–5 (*n* = 130)	17 (13.1)	1	0.855	1	0.804
6–10 (*n* = 137)	20 (14.6)	1.13 (0.56–2.27)		1.09 (0.53–2.24)	
11–15 (*n* = 82)	24 (29.3)	2.75 (1.36–5.52)		2.38 (1.15–4.94)	0.018

*Gender*					
Male (*n* = 179)	38 (21.2)	1.72 (0.97–3.03)	0.079	1.59 (0.88–2.89)	0.123
Female (*n* = 170)	23 (13.5)	1		1	

*Poverty*					
Yes (*n* = 249)	48 (19.4)	1.60 (0.82–3.11)	0.102	1.23 (0.60–2.50)	0.559
No (*n* = 100)	13 (13)	1		1	

*Albendazole in the last six months*					
Yes (*n* = 120)	15 (12.5)	0.56 (0.30–1.06)	0.104	0.56 (0.29–1.09)	0.091
No (*n* = 229)	48 (20.1)	1		1	

*Open evacuation*					
Yes (*n* = 143)	35 (24.5)	2.24 (1.28–3.93)	0.006	1.02 (0.46–2.23)	0.958
No (*n* = 206)	26 (12.6)	1		1	

*Living in stilt houses*					
Yes (*n* = 83)	19 (22.4)	1.58 (0.86–2.91)	0.186	1.21 (0.60–2.44)	0.584
No (*n* = 266)	42 (15.8)	1		1	

*Latrine inside house*					
Yes (*n* = 166)	16 (9.6)	0.32 (0.17–0.60)	<0.001	0.38 (0.17–0.85)	0.019
No (*n* = 183)	45 (24.6)	1		1	

*Trichuris trichiura coinfection*					
Yes (*n* = 127)	49 (38.6)	10.99 (5.55–21.75)	<0.001	NP	NP
No (*n* = 222)	12 (5.4)	1			

*Hookworms coinfection*					
Yes (*n* = 26)	17 (65.4)	11.93 (5.02–28.53)	<0.001	NP	NP
No (*n* = 323)	44 (13.6)	1			

**Table 2:** Factors associated with trichuriasis in children living in the urban area of Bagre, Pará State, Brazilian Amazon.

	Positive for *T. trichiura*, *N* (%)	Crude OR (95% CI)	*p* value	Adjusted OR (95% CI)	*p* value
*Age group (years)*					
0–5 (*n* = 130)	27 (20.8)	1		1	
6–10 (*n* = 137)	60 (43.8)	2.97 (1.72–5.10)	<0.001	3.31 (1.85–5.89)	<0.001
11–15 (*n* = 82)	40 (48.8)	3.63 (1.98–6.65)	<0.001	3.16 (1.66–6.00)	<0.001

*Gender*					
Male (*n* = 179)	73 (40.8)	1.47 (0.95–2.29)	0.101	1.44 (0.89–2.34)	0.132
Female (*n* = 170)	54 (31.8)	1			

*Poverty*					
Yes (*n* = 249)	101 (40.7)	1.95 (1.17–3.26)	0.013	1.78 (1.01–3.14)	0.045
No (*n* = 100)	26 (26)	1		1	

*Albendazole in the last six months*					
Yes (*n* = 120)	37 (30.8)	0.68 (0.43–1.10)	0.148	0.62 (0.37–1.04)	0.073
No (*n* = 229)	90 (39.3)	1		1	

*Open evacuation*					
Yes (*n* = 143)	73 (51)	2.93 (1.86–4.61)	<0.001	2.07 (1.07–3.99)	0.029
No (*n* = 206)	54 (26.2)	1		1	

*Living in stilt houses*					
Yes (*n* = 83)	35 (42.2)	1.37 (0.83–2.28)	0.131	0.74 (0.40–1.36)	0.339
No (*n* = 266)	92 (34.6)	1			

*Latrine inside house*					
Yes (*n* = 166)	39 (23.5)	0.33 (0.20–0.52)	<0.001	0.55 (0.29–1.05)	0.071
No (*n* = 183)	88 (48.1)	1		1	

*Ascaris lumbricoides coinfection*					
Yes (*n* = 61)	49 (80.3)	10.99 (5.55–21.75)	<0.001	NP	NP
No (*n* = 288)	78 (27.1)	1			

*Hookworms coinfection*					
Yes (*n* = 26)	20 (76.9)	6.72 (2.62–17.2)	<0.001	NP	NP
No (*n* = 323)	107 (33.1)	1			

**Table 3:** Factors associated with hookworm infection in children living in the urban area of Bagre, Pará State, Brazilian Amazon

	Positive for hookworms, *N* (%)	Crude OR (95% CI)	*p* value	Adjusted OR (95% CI)	*p* value
*Age group (years)*					
0–5 (*n* = 130)	4 (3.1)	1		1	
6–10 (*n* = 137)	8 (5.8)	1.95 (0.57–6.65)	0.427	2.03 (0.55–7.45)	0.285
11–15 (*n* = 82)	14 (17.1)	6.48 (2.05–20.47)	<0.001	6.70 (1.91–23.43)	0.002

*Gender*					
Male (*n* = 179)	22 (12.3)	5.81 (1.96–17.25)	<0.001	6.35 (2.00–20.14)	0.002
Female (*n* = 170)	4 (2.4)	1			

*Poverty*					
Yes (*n* = 249)	25 (10.1)	11.09 (1.48–83.6)	0.007	7.66 (0.97–60.35)	0.053
No (*n* = 100)	1 (1)	1		1	

*Albendazole in the last six months*					
Yes (*n* = 120)	5 (4.2)	0.43 (0.15–1.17)	0.139	0.31 (0.10–0.96)	0.042
No (*n* = 229)	21 (9.2)	1		1	

*Open evacuation*					
Yes (*n* = 143)	16 (11.2)	2.46 (1.08–5.61)	0.044	0.58 (0.16–2.11)	0.417
No (*n* = 206)	10 (4.9)	1		1	

*Living in stilt houses*					
Yes (*n* = 83)	13 (15.7)	3.61 (1.60–8.14)	0.002	3.52 (1.22–10.12)	0.019
No (*n* = 266)	13 (4.9)	1		1	

*Latrine inside house*					
Yes (*n* = 166)	6 (3.6)	0.30 (0.11–0.78)	<0.016	0.39 (0.10–1.42)	0.155
No (*n* = 183)	20 (10.9)	1		1	

*Ascaris lumbricoides coinfection*					
Yes (*n* = 61)	17 (27.9)	11.97 (5.02–28.5)	<0.001	NP	NP
No (*n* = 288)	9 (3.1)	1			

*Trichuris trichiura coinfection*					
Yes (*n* = 127)	20 (15.7)	6.72 (2.62–17.24)	<0.001	NP	NP
No (*n* = 222)	6 (2.7)	1			

## Data Availability

Data are available on request due to privacy/ethical restrictions. The data that support the findings of this study are available on request to the corresponding author, Calegar DA. The data are not publicly available to protect the privacy of research participants.
